# Sexual Dimorphism Has Low Impact on the Response against Rotavirus Infection in Suckling Rats

**DOI:** 10.3390/vaccines8030345

**Published:** 2020-06-29

**Authors:** Carla Morales-Ferré, Ignasi Azagra-Boronat, Malén Massot-Cladera, Àngels Franch, Margarida Castell, María José Rodríguez-Lagunas, Francisco J. Pérez-Cano

**Affiliations:** 1 Physiology Section, Department of Biochemistry and Physiology, Faculty of Pharmacy and Food Science, University of Barcelona (UB), 08028 Barcelona, Spain; carla.moralesferre@ub.edu (C.M.-F.); ignasiazagra@ub.edu (I.A.-B.); malen.massot@ub.edu (M.M.-C.); angelsfranch@ub.edu (À.F.); margaridacastell@ub.edu (M.C.); franciscoperez@ub.edu (F.J.P.-C.); 2 Nutrition and Food Safety Research Institute (INSA-UB), 08921 Santa Coloma de Gramenet, Spain

**Keywords:** rotavirus, dimorphism, immunity, diarrhea

## Abstract

Rotaviruses (RVs) are the leading pathogens causing severe and acute diarrhea in children and animals. It is well known that sex contributes to shaping immune responses, thus it could also influence the incidence and severity of the RV infection. The aim of this study was to analyze the influence of sexual dimorphism on RV infection and its antibody (Ab) immune response in a suckling rat model. Neonatal suckling rats were intragastrically RV-inoculated and clinical indexes derived from fecal samples, as well as immune variables were evaluated. Higher severity of diarrhea, fecal weight and viral elimination were observed in males compared to females (*p* < 0.05). Regarding the adaptative immunity, the RV shaped the immune response to lower IgG1 levels and an increased Th1/Th2-associated Ab response (*p* < 0.05). Although females had lower IgG2a levels than males (*p* < 0.05), the specific anti-RV antibody levels were not sex influenced. In fact, at this age the passive transfer of anti-RV antibodies through breast milk was the critical factor for clustering animals, independently of their sex. It can be concluded that male and female diarrhea severity in RV infection is slightly influenced by sexual dimorphism and is not associated with the specific immune response against the virus.

## 1. Introduction

It is widely accepted that males and females have differences in their immune system. Females generally show stronger humoral and cellular immune responses to infection or antigenic stimulation than males, which can provide protection against pathogens but can lead to an aberrant inflammatory response increasing the risk of autoimmune diseases [1,2,3,4].

Several studies have documented the gender-specific differences in the infection rate of certain bacterial, parasitic and viral infective agents, such as *Mycobacterium tuberculosis*, Plasmodium parasites, West Nile virus, human immunodeficiency virus and several hantaviruses, with males being those with the highest susceptibility to these infections [4]. Moreover, there are also sex differences in the severity of some viral infections [4,5,6]. For instance, some studies have demonstrated that women suffering from acute infection of Hepatitis C virus are more likely to spontaneously eliminate the virus. In addition, during the chronical stage of this infection, women have less risk of cirrhosis compared to men [7].

The mechanisms for these sexual differences are diverse, and include genetic, epigenetic, hormonal and environmental factors [4,5,8]. Different immunological responses observed between sexes are attributed to the biased response derived from the X chromosome, which contains a large number of immune-related genes that encode receptors, microRNAs and transcription factors involved in the regulation of the immune system [5]. In addition, sex-specific epigenetic mechanisms such as DNA methylation and chromatin remodeling also modulate the immune system [6]. In this regard, sexual hormones like estrogens, progesterone and testosterone quantitatively and qualitatively modulate the responses of immune cells [6,8,9]. Moreover, several studies have reported that gut microbiota composition differs from males to females [10,11,12], which in turn can influence the immune system. Eventually, all the above differences could have an influence on the degree of protection in vaccination.

Rotavirus (RV) is a non-enveloped virus of the family Reoviridae with a double-stranded RNA genome that infects enterocytes of the small intestine. Its replication in the duodenal mucosa of infants causes disruption to cellular homeostasis resulting in enterocyte damage and cell death [13,14,15]. RV is the leading causative agent of acute diarrhea in children and is responsible for approximately 20−30% of all cases of severe gastroenteritis that require treatment in hospitals [14,16]. The severity of RV diarrhea is associated with dehydration, as well as other risk factors, such as nutritional status, age under two years, and early complementary feeding [15,17,18].

The use of animal models of RV infection has allowed us to understand the pathogenesis of this infection, the immune response and to test vaccine efficacy [14,15,19]. The RV infection model in rodents has been widely used to study the diarrhea process due to short gestations and multiparous births, which allow us to incorporate large numbers of animals in a short period of time in the studies [20,21,22,23,24]. Even though there are many studies about the different susceptibility and severity for infections in each sex, to our knowledge, there is almost no information on the gender-specific differences in RV infection. For this reason, this study aimed to determine the differences between sexes on the incidence and severity of diarrhea and the subsequent immune response in an RV-infected neonatal rat model.

## 2. Materials and Methods 

### 2.1. Animals

A total of 23 Lewis pregnant rats obtained from Janvier Labs (Le Genest-Saint-Isle, France) and Harlan (Barcelona, Spain) were used. Animals included in this study were derived from previous independent studies [20,21,25,26], allowing enough power analysis to draw conclusions. In all cases, gestating rats were housed individually in cages, monitored daily and allowed to deliver at term. The day of birth was registered as day 1 of life. Litters were unified to the same number of pups per lactating dam based on the original study, with free access to the nipples and rat diet. The dams were fed with a commercial diet corresponding to the AIN-93G formulation [27] and given mineral water ad libitum. Animal handling was performed during the first hours of the light phase on a scheduled basis to limit the disturbance and influence of biological rhythms. The animals were housed under controlled temperature and humidity conditions, in a 12:12-h light/dark cycle. They were located in a special safe and isolated room designed and authorized for working under biosecurity level 2 conditions at the Animal Service of the Faculty of Pharmacy and Food Science, in the University of Barcelona. All experimental procedures were conducted in accordance with the institutional guidelines for the care and use of laboratory animals and were approved by the Ethical Committee for Animal Experimentation of the University of Barcelona and the Catalonia Government (DAAM9268, DAAM3406 and DAAM10176), in full compliance with national legislation following the EU-Directive 2010/63/EU for the protection of animals used for scientific purposes.

### 2.2. Experimental Design

Upon natural delivery, pups were distributed into two groups: the reference (REF) group and the RV-infected (RV) group. Body weight was recorded daily throughout the study to assess weight gain.

The simian SA-11 RV strain was obtained from the group Virus Entèrics from the University of Barcelona (Dr. A. Bosch and Dr. R. Pintó). Viruses were propagated in fetal African green monkey kidney cells (MA-104) and titered as TCID50/ mL (TCID, tissue culture infection dose) [23]. SA-11 was intragastrically inoculated (∼2 × 10^8^ TCID_50_ RV/rat) in 100 µL of phosphate buffered saline (PBS) to suckling rats at days 5–7 of life, depending on the initial study, as previously described [23]. The RV was inoculated after 1 h of separation from their dams to avoid interference between the RV and milk components.

Clinical evaluation was performed daily from the day before inoculation (considered in all studies as day −1 day post-inoculation—DPI) until the end of the study 11 days later (11 DPI). Animals were euthanized at two different time points: a group of the animals was euthanized on 3 DPI (n = 42), whereas the others were euthanized at 11 DPI (n = 139). To analyze the data, rats were divided into 4 groups taking into consideration their sex (male REF (n = 39), female REF (n = 46), male RV (n = 39), and female RV (n = 57) groups). From each original study, the number of males and females in both REF and RV groups included a similar number of pups.

### 2.3. Clinical Indexes and Fecal Specimen Collection

SA-11 infection was evaluated from day −1 to 11 DPI by the growth rate and clinical indexes derived from fecal samples. Growth rate was expressed as body weight increase calculating the difference in weight during the pre-diarrhea period (from −3 DPI to 0 DPI), diarrhea period (from 1 DPI to 5 DPI) and post-diarrhea period (from 6 DPI to 9 DPI). Collection of individual fecal samples was performed daily by gently pressing and massaging the abdomen. Specimens were immediately scored, weighed and frozen for further analysis. The severity of diarrhea was expressed by the fecal weight and by scoring stools from 1 to 4 (diarrhea index (DI)) as described: normal (1), loose yellow-green (2), totally loose yellow-green (3), and high amount of watery (4) feces. Diarrhea scores ≥ 2 indicate diarrheic feces, whereas scores of DI < 2 indicate absence of diarrhea [23].

The area under the curve of severity (sAUC) from 0 to 6 DPI—the last day with signs of diarrhea in some animals—was calculated as a global value of severity. The maximum diarrhea index (MDI) was defined as the highest score during the diarrhea period. Incidence of diarrhea was expressed as the percentage of diarrheic animals (% DA, consisting of the percentage of animals presenting diarrhea in each group) and by percentage of diarrheic feces (% DF, taking into consideration the number of total samples collected every day in each group). The AUCs of % DA and % DF (daAUC and dfAUC) during 0–6 DPI were calculated as global values of incidence. The maximum percentage of diarrheic animals (MDA) and diarrheic feces (MDF) were defined as the highest values during the diarrhea period. The days when MDI, MDA and MDF were achieved were also used as indicators (MDId, MDAd and MDFd, respectively). The diarrhea period (DP) was calculated for each animal as the interval between the first (day of diarrhea beginning, DDB) and the last day (day of diarrhea ending, DDE) of diarrhea. The actual number of days with diarrhea within the diarrhea period were also counted (days with diarrhea, DwD).

### 2.4. Fecal SA-11 Shedding

Fecal samples from 1 DPI were diluted in PBS (up to 10 mg/mL) and homogenized using Pellet Pestles Cordless Motor (Sigma-Aldrich, Madrid, Spain). Homogenates were centrifuged (170 g, 5 min, 4 °C) and supernatants were frozen at −20 °C until use. SA-11 particles were quantified by ELISA, as previously described [23]. Titrated dilutions of SA-11 particles, ranging from 10^6^ to 10^4^/mL, were used for the standard curve. Data were expressed as SA-11 particles/mg fecal sample. In addition, the total RV in feces was calculated multiplying the concentration of RV and the weight of the feces from 1 DPI.

### 2.5. Sample Collection and Processing

At day 3 DPI or 11 DPI, animals were intramuscularly anesthetized with ketamine (90 mg/kg) (Merial Laboratories S.A., Barcelona, Spain) and xylazine (10 mg/kg) (Bayer A.G., Leverkusen, Germany). On those days, collected blood samples were used to determine the immunoglobulin (Ig) pattern and anti-RV antibodies (Ab) from plasma. Small intestine was excised and a 0.5 cm portion of the distal jejunum was immediately conserved in RNAlater^®^ (Ambion, Applied Biosystems, Austin, TX, USA), incubated at 4 °C overnight and stored at −20 °C until PCR analysis. The remaining part of the small intestine was opened lengthwise, cut into 5 mm pieces, incubated with 2 mL of PBS in a shaker (10 min, 37 °C) and then centrifuged in order to obtain the gut wash (GW), which enabled the quantification of the intestinal permeability. Furthermore, at 11 DPI the stomach content of exclusive suckling rats was used as equivalent sample of their corresponding mother’s breast milk and was used to study the passive transfer of anti-RV antibodies. 

### 2.6. Anti-RV Antibodies in Plasma

Anti-RV IgM and total anti-RV Ab in pups’ and dams’ plasma were quantified by ELISA. A fraction of frozen stomach content from rats at 11 DPI was homogenized and diluted in PBS and tested for the presence of anti-RV Ab. Following previous procedures [23,26], UV-inactivated SA-11 particles at 10^5^/mL were used for coating the 96-well plates (Nunc MaxiSorp, Wiesbaden, Germany). After blocking with 1% bovine serum albumin (BSA in PBS) for 1 h at room temperature (RT), appropriate diluted plasma (1/6) was added (3 h, RT). Biotin anti-rat IgM (BD Biosciences, Heidelberg, Germany) was added for measuring anti-RV IgM and goat peroxidase-conjugated anti-rat IgG, IgA and IgM (H + L) (Sigma-Aldrich, Madrid, Spain) for 1 h each were added for measuring total anti-RV. For measuring IgM, extravidin-peroxidase conjugate (4 µg/mL in PBS-Tween-1% BSA; Sigma–Aldrich, Madrid, Spain) was added for 30 min at RT. Subsequently, in both cases, substrate solution (o-phenylenediamine plus hydrogen peroxide in 0.2 M phosphate, 0.1 M citrate buffer, pH 5; Sigma-Aldrich, Madrid, Spain) was added. Absorbance was measured at 492 nm after stopping the enzymatic reaction with 3 M H_2_SO_4_ on a microtiter plate photometer (Labsystems, Helsinki, Finland). Data were interpolated by means of Multiskan Ascent v.2.6 software (Thermo Fisher Scientific SLU, Barcelona, Spain). Pooled serum from dams of inoculated litters was used as a standard in each plate. Dilutions of dam serum ranged from 1/80 to 1/5120 and from 1/10 to 1/800 to measure IgM and total anti-RV Ab, respectively. Results were normalized giving RV females of each experiment the value of 100%.

### 2.7. Immunoglobulins in Plasma

Plasma concentrations of IgG1, IgG2a, IgG2b, IgG2c, IgM and IgA were quantified on 11 DPI using ProcartaPlexTM Multiplex immunoassay (eBioscience, San Diego, CA, USA) as in previous studies [21], in which, specific color-coded capture beads were bound to the Ig of interest. Then, different detection antibodies conjugated to phycoerythrin (PE) were added. The specific concentration of each analyte was obtained by MAGPIX^®^ analyzer (Luminex Corporation, Austin, TX, USA) at the Scientific and Technological Centers of the University of Barcelona (CCiT-UB). The sensitivity of the assay was as follows: 0.02 ng/mL for IgM; 0.78 ng/mL for IgG1; 0.02 ng/mL for IgG2a; 0.11 ng/mL for IgG2b; 0.19 pg/mL for IgG2c and 0.48 pg/mL for IgA.

### 2.8. Intestinal Permeability

The quantification of α1-antitrypsin (A1AT) in the gut wash, as a marker of intestinal permeability, was performed with the rat SERPINA1/Alpha 1 Antitrypsin ELISA kit (LifeSpan Biosciences Inc., Seattle, WA, USA) following the manufacturer’s instructions as in previous studies [21]. The standard concentrations ranged from 100 to 1.563 ng/mL. Assay sensitivity was 1.56 ng/mL. Results were normalized giving REF females of each experiment the value of 100%.

### 2.9. Gene Expression Analysis

A 0.5 cm portion of the central section of the small intestine of 3-DPI pups (during the peak of disease) and of 11-DPI rats (after the diarrhea was resolved) were homogenized for 30 s in lysing matrix tubes (MP Biomedicals, Illkirch, France) using a FastPrep-24 instrument (MP Biomedicals), as previously described [21]. After RNA isolation with the RNeasy^®^ Mini Kit (Qiagen, Madrid, Spain) its purity and concentration was determined with a NanoPhotometer (BioNova Scientific S.L., Fremont, CA, USA) and the corresponding cDNA was obtained using a thermal cycler PTC-100 Programmable Thermal Controller and TaqMan^®^ Reverse Transcription Reagents (Applied Biosystems, AB, Weiterstadt, Germany).

The specific PCR TaqMan^®^ primers (AB) used to assess gene expression with real-time PCR (ABI Prism 7900 HT, AB) were directed to the detection of barrier function molecules such as Muc2 (Rn01498206_m1, inventoried (I)), Ocln (Rn00580064_m1, I) and Cldn2 (Rn02063575_s1, I) as well as to toll-like receptors (TLR), such as Tlr2 (Rn02133647_s1, I), Tlr4 (Rn00569848_m1, I), Tlr5 (Rn04219239_s1, I), Tlr7 (Rn01771083_s1, I) and Tlr9 (Rn01640054_m1, I). The relative gene expression was normalized to the housekeeping gene Gusb (Rn00566655_m1, I) using the 2-ΔΔCt method. Results were expressed as the percentage of expression in each experimental group normalized to the mean value obtained for the female REF group of each experiment, which was set at 100%.

### 2.10. Statistical Analysis

Results are expressed as mean ± SEM. The Statistical Package for Social Sciences (SPSS v22.0) (IBM, Chicago, IL, USA) was used for statistical analysis. Data were tested for homogeneity of variance and normality distribution by Levene’s and Shapiro-Wilk tests, respectively. When data were homogeneous and had a normal behavior, a conventional one-way ANOVA test was performed. Otherwise, the nonparametric Kruskal-Wallis test followed by the post hoc Mann-Whitney U test was carried out. The chi-square test was performed to compare frequencies of DA and DF. Finally, the Spearman correlation coefficient was used to search for correlation between weight and clinical variables and between immunological variables. Significant differences were established when *p* < 0.05.

A principal components analysis (PCA) was performed with Simca v14.1 (Umetrics, Umea, Sweden) to analyze the natural clustering of samples. Two data matrices were constructed consisting of 96 rows and 12 variables (DI on 1-5 DPI, MDI, MDId, sAUC, DP, DwD, DDE and DDB), in order to analyze the variance observed in the clinical indicators of diarrhea, and 21 rows and 15 variables (total anti-RV Ig, anti-RV IgM, IgG, IgG1, IgG2a, IgG2b, IgG2c, total Ig, % IgM, % IgA, % IgG, Th1/Th2 and RV shedding), in order to analyze the variance observed in the anti-RV response. In the preprocessing of the PCA, Pareto scaling was applied. Data were represented in score and loading plots.

## 3. Results

### 3.1. Body Weight

**Figure 1:**
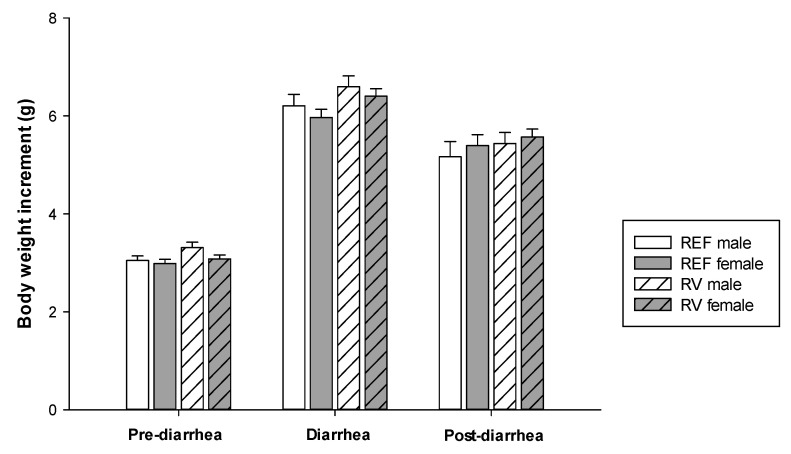
Body weight gain (g) during the pre-diarrhea period (from −3 DPI to 0 DPI), diarrhea period (from 1 DPI to 5 DPI) and post-diarrhea period (from 6 DPI to 9 DPI). Results are expressed as mean ± SEM (n = 39–57 animals/group).

Body weight (BW) was recorded between days 2 and 16 of life. To analyze the possible effect of viral infection on the BW, the increase in BW gain was calculated during the pre-diarrhea (from −3 DPI to 0 DPI), diarrhea (from 1 DPI to 5 DPI) and post-diarrhea (from 6 DPI to 9 DPI) periods (Figure 1). Although RV infection causes intestinal malabsorption, with associated fluid loss and dehydration inducing BW loss, in our study no changes in BW were observed. Thus, similar BW gain during the diarrhea period was observed for both sexes and also during the post-diarrhea period. Overall, BW was not influenced by sex in the context of RV infection.

### 3.2. Incidence of Diarrhea

**Figure 2:**
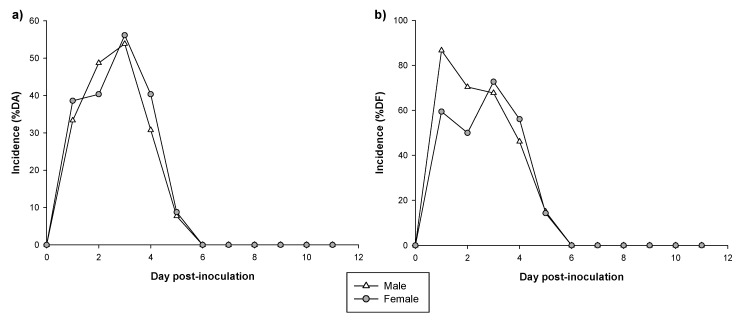
Incidence of RV infection expressed as percentage of diarrheic animals (% DA) (**a**) and diarrheic faces (% DF) (**b**) from 0 DPI to 11 DPI in RV infected group (n = 39–57 animals/ group).

The incidence of RV-induced diarrhea was evaluated using two approaches: the percentage of diarrheic animals (% DA) and the percentage of diarrheic feces (% DF) (Figure 2). Looking at the % DA during the whole period, most of the animals (85% of males and 89% of females) displayed diarrhea at some time point without differences between sexes. 

Specifically, as can be observed in Figure 2a, the %DA in males was 33% on 1 DPI. It increased to 49% on 2 DPI reaching the maximum incidence on 3 DPI (54%). From 4 DPI to 5 DPI it decreased to 31% and 8%, respectively. On 6 DPI none of the males had diarrhea. The %DA in females was similar to males: the %DA was 39% on 1 DPI, remained constant on 2 DPI (40%), increased to 56% on 3 DPI and decreased from 4 DPI to 5 DPI (40% and 9% respectively). On 6 DPI none of the females had diarrhea (Figure 2a).

Alternatively, the %DF in males was 87% on 1 DPI. It decreased to 70% on 2 DPI, the incidence was similar on 3 DPI (68%) and almost half of males had diarrhea on 4 DPI. Later, on 5 DPI, only 15% of males still had some diarrhea and, on 6 DPI, none of the males had diarrhea (Figure 2b). When the diarrhea incidence was studied in females, this group showed a tendency to have lower %DF on 1 DPI (59%) and 2 DPI (50%) compared to males (87% and 70%, respectively), but without statistical significance (*p* = 0.06 and *p* = 0.09, respectively). On 3 DPI it increased to 73%, reaching the maximum %DF. On 4 and 5 DPI it decreased to 56% and 14%, respectively. Later, on 6 DPI, as occurred in the males, none of the females had diarrhea (Figure 2b). 

If we focus on the maximum incidences found and the days they were obtained (Table 1), both groups achieved similar MDA percentages on 3 DPI. However, males had the MDF one day after the induction whereas females reached similar characteristics of MDF three days after induction (*p* < 0.05). No differences were observed in the dfAUC and daAUC between sexes.

Focusing on the duration of the diarrhea process, in both groups, diarrhea started 1–2 days after inoculation (DDB) and ended around 3 DPI (DDE) but no differences were observed in the DP and DwD between sexes (Table 1).

### 3.3. Severity of Diarrhea

**Figure 3:**
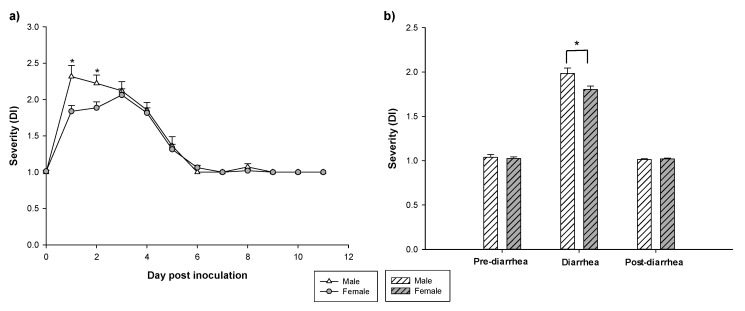
Severity of diarrhea along the study (**a**), and grouped in the pre-diarrhea, diarrhea and post-diarrhea periods (**b**). The pre-diarrhea last from −3 DPI to 0 DPI, diarrhea from 1 DPI to 5 DPI and post-diarrhea period from 6 DPI to 11 DPI. Results are expressed as mean ± SEM (n = 39–57 animals/group). Statistical differences: *: *p* < 0.05 males vs. females.

Overall, males had a higher sAUC compared to females (*p* < 0.05, Table 1). As can be observed in Figure 3a, the mean severity on 1 and 2 DPI was higher in males compared to females (*p* < 0.05). After that, on 3 DPI, the mean severity in both sexes was similar in males and females and at 4 DPI, the mean score in both sexes was under two showing a similar reduction pattern and meaning that most animals did not have diarrhea.

**Figure 4:**
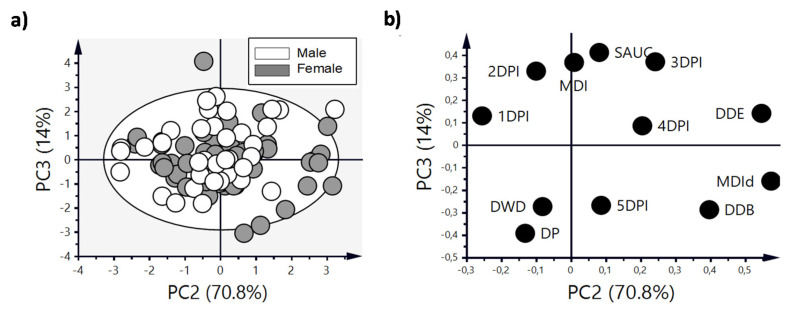
Principal components analysis of the clinical variables of diarrhea. (**a**) Scores plot graph, showing the natural clustering of samples and (**b**) loadings plot, showing how the variables contribute to the variance. Results are derived from n = 96 animals (57 females and 39 males).

The mean DI corresponding to the three periods (pre-diarrhea, diarrhea and the post-diarrhea periods) allows a better observation of this difference. It shows that males had a higher mean severity during the diarrhea period compared to females (*p* < 0.05) (Figure 3b). If we focus on the maximum diarrhea index (Table 1), a higher MDI was observed in males compared to females (*p* < 0.05). In addition, the day of maximum diarrhea index (MDId, Table 1) was achieved earlier in males than in females (*p* < 0.05). The PCA showed that the natural clustering of RV-infected animals overlapped between males and females (Figure 4).

However, a subgroup of females clustered on the right part of the scores plot (Figure 4a) and these were characterized mainly by achieving a later MDId (Figure 4b).

### 3.4. Fecal Weight

**Figure 5:**
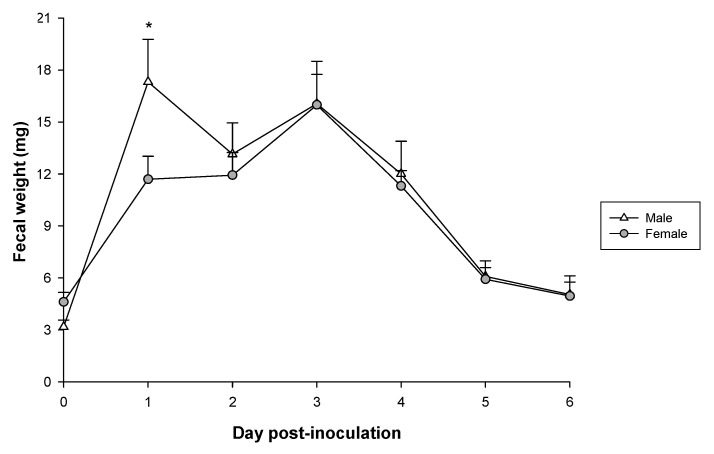
Fecal weight (mg) from 0 DPI to 6 DPI in RV infected group. Results are expressed as mean ± SEM (n = 10–38 samples/group, depending on the number of fecal samples obtained each day). Statistical differences: *: *p* < 0.05 males vs. females.

Fecal weight was also measured as an objective severity variable of the diarrheic process (Figure 5). Before the induction of RV infection, the fecal weight among groups was similar. A statistically significant increase of 48% in fecal weight was observed in males on 1 DPI compared to females (*p* < 0.05). Later, it decreased in males on 2 DPI and increased on 3 DPI, whereas in females, fecal weight increased on 2 DPI and reached its highest value on 3DPI. From 4 DPI to 6 DPI the fecal weight progressively decreased in both sexes without significant differences. 

We observed a positive correlation between animal weight and the fecal weight on 1 DPI in both RV (R = 0.444, *p* < 0.05) and REF groups (R = 0.306, *p* < 0.05), but not on 0 DPI. When clustering the animals depending on the sex, this correlation was only observed in the females of the RV group (R = 0.381, *p* < 0.05). Moreover, in all cases the fecal weight displayed high correlation with the DI (R = 0.743 in females and R = 0.807 in males, *p* < 0.05).

### 3.5. Fecal SA-11 Shedding and Intestinal Barrier Function 

**Figure 6:**
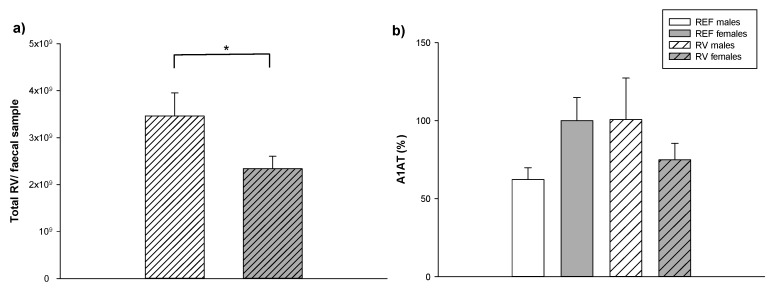
Viral shedding in feces from animals in the RV group and assessment of intestinal barrier function in both, REF and RV groups. The viral shedding was assessed at the peak of viral elimination (1 DPI). Results are represented by and total RV/ fecal sample (**a**). The percentage of alpha-1 antitrypsin (A1AT) concentration in the gut wash (**b**) was analyzed as a measure of the intestinal barrier disruption. Results are expressed as mean ± SEM (n = 39–57 samples/group). Statistical differences: *: *p* < 0.05 RV males vs. RV females.

The viral shedding in feces (Figure 6a) was quantified by ELISA at the day of maximum elimination, 1 DPI [27]. Although, both sexes eliminated 2–3 × 10^6^ RV particles/mg of feces without differences between sexes, the total amount of RV cleared by fecal sample from 1 DPI was higher in males than in females (*p* < 0.05).

In addition, the A1AT levels in the gut (Figure 6b), as an indicator of intestinal permeability, were measured by ELISA on 3 DPI. During the inflammatory response, the levels of A1AT in plasma are elevated and can be transported into the intestinal lumen due to the increase in the permeability of the intestinal barrier (21). There were no statistically significant differences between sexes either in the REF or the RV groups. However, the males, but not the females, showed a tendency (*p* = 0.07) to have a 1.5 increase in the levels of A1AT in the gut wash.

### 3.6. Intestinal Gene Expression 

**Figure 7:**
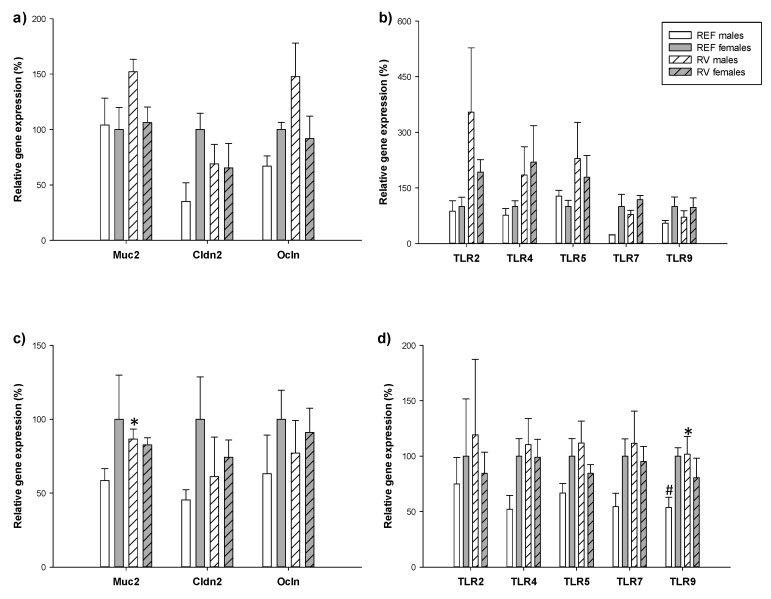
Relative expression of mucin (Muc2). tight junction molecules claudin (Cldn2) and occludin (Ocln), and toll like receptors (TLR) 2, TLR4, TLR5, TLR7 and TLR9 was quantified by real-time PCR on 3 DPI (**a**,**b**) and 11 DPI (**c**,**d**). Relative gene expression was calculated with respect to REF females, which corresponded to 100% of transcription. Results are expressed as mean ± S.E.M. (n = 3–6 animals/group). * *p* < 0.05 vs. non-infected matched group; ^#^ *p* < 0.05 vs. female matched group.

To ascertain the involvement of the barrier function proteins and signaling receptors involved in cross-talking in the sex-differential clinical outcomes the gene expression of mucin, claudin 2 (Cldn2), occludin (Ocln) and toll-like receptors (TLRs) was measured on 3 and 11 DPI (Figure 7).

On 3 DPI, the gene expression of molecules involved in barrier integrity and bacteria host crosstalk was similar between sexes in basal conditions (REF group). However, REF females showed a tendency to have higher levels of Cldn2, Ocln and TLR7 (×2 times) compared to the REF males (*p* = 0.06 in all cases). In the context of RV infection, males had a tendency to increase the expression of the same genes and also TLR4 (×1.5–2 times) in comparison to REF males (*p* = 0.08 in all cases). Without being statistically significant, RV females’ levels of TLR2 and TLR4 were two times higher than those of REF females (*p* = 0.05 and *p* = 0.09, respectively). No differences were observed in the expression of TLR5 and TLR9 (Figure 7a,b).

On 11 DPI, the only difference between sexes in REF conditions was found for TLR9, with the levels in females being two times higher than in males (*p* < 0.05). In the RV group, the gene expression of Muc2 and TLR9 in males was 1.5–2 times higher than in REF males (*p* < 0.05) and showed a tendency to increase TLR4 and TLR7 levels (*p* = 0.08). No differences were observed in the gene expression of Cldn2, Ocln, TLR2 and TLR5 between groups. (Figure 7c,d). 

### 3.7. Antibody Production

Plasma concentrations of IgG, IgM and IgA isotypes, IgG subclasses, as well as the Th1/Th2 ratio were quantified on 11 DPI, once the infection was resolved. As can be observed in Table 2, there was no difference associated with sex in noninfected animals (REF males vs. REF females). In line with this, RV males and RV females at this early age showed a similar concentration of IgG1, IgG2a, IgG2b, IgG2c, total IgG, IgA and IgM and a similar Th1/Th2 ratio. However, the immune response against the virus led to a decrease in the concentration of IgG1 (Th2-associated subclass) in both RV males and RV females compared to their non-infective groups (*p* < 0.05). This change led to shaping the Ig pattern to a more Th1 response in the RV groups as shown by a higher Th1/Th2 ratio (*p* < 0.05). In addition, RV females showed a reduction in IgG2a (Th2-associated subclass) compared to REF females (*p* < 0.05), whereas in males this change was not statistically significant. 

**Figure 8:**
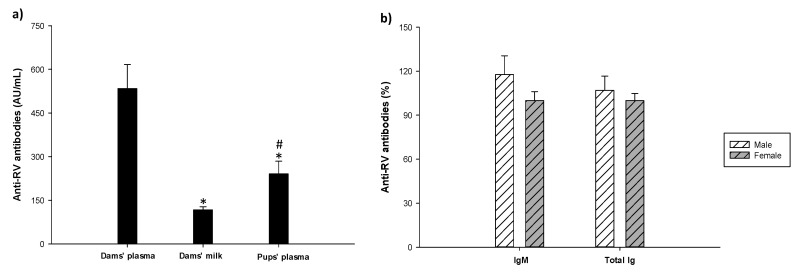
Concentration of total anti-RV antibodies in dams’ plasma, milk and pups’ plasma expressed in Arbitrary Units/mL (**a**) and anti-RV antibodies (IgM and total Ig) in plasma of male and female rats at the end of the study (11 DPI) in the RV group (**b**). Results are expressed as mean ± S.E.M (n = 16–30 animals/group). Statistical differences: * *p* < 0.05 vs. dams’ plasma; ^#^ *p* < 0.05 vs. milk.

Anti-RV antibodies were measured in pregnant dams’ plasma and in plasma and the milk from the stomach of pups on 11 DPI. The total anti-RV antibodies in dams’ plasma had the highest levels, followed by those in pups’ plasma, and then in milk (*p* < 0.05) (Figure 8a). 

In order to explore the anti-RV passive transfer from dams to pups in more depth, the correlation of total anti-RV antibodies concentration between dams’ milk, dams’ plasma and pups’ plasma was investigated. Although no correlation coefficient was observed between dams’ plasma and milk (R = 0.09 and *p* = 0.78), a positive correlation was observed between dams’ milk and pups’ plasma (R = 0.45 and *p* < 0.05), indicating the role of milk Ab transfer at this stage of life. Focusing on pups’ plasma, no differences were observed between sexes in total or IgM anti-RV antibodies in pups’ plasma (Figure 8b).

**Figure 9:**
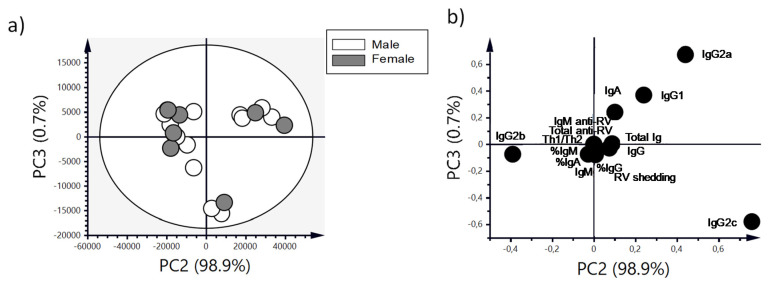
Principal components analysis of variables of the anti-RV response. Scores plot graph, showing the natural clustering of samples (**a**) and loadings plot, showing how the variables contribute to the variance (**b**). Results are derived from n = 21 animals (14 females and 7 males).

Upon PCA analysis taking together both types of data (Ig subtypes/subclasses and anti-RV Ab levels, Figure 9), the score’s plot revealed that the natural clustering of the infected animals showed three different groups, although none of them showed separation of males and females (Figure 9a). In addition, we identified that the samples of siblings (sharing the same mother) always clustered together. In fact, most of the variance of the samples, as observed in the loading plot (Figure 9b), was determined by IgG2a (upper-right cluster) or IgG2b and IgG2c (bottom-left and -right clusters, respectively).

## 4. Discussion 

Many reports document a wide variation in the susceptibility to disease and in the immune response between males and females during viral infection due to genetic, epigenetic, hormonal and environmental factors [4,5,8]. Viral gastroenteritis is frequently associated with contaminated food or water. The main causative pathogens worldwide are RVs but also norovirus pathogens and in lower proportions some others such as adenovirus types 40 and 41, astrovirus, calicivirus, and small round structured viruses [28]. However, taking into consideration that the infections are mild and self-limiting there is no need to specifically identify the etiological agent. Therefore, it is difficult to clinically distinguish the etiological agent as well as the sex response to it. Thus, having a controlled animal model to study sex differences in the immune response to a specific pathogen is necessary. In the case of the RV infection the susceptibility and severity of RV infection based on sex differences has not been thoroughly studied. For this reason, a rat neonatal RV gastroenteritis model was used herein.

One of the main features of human RV infection is the consequent diarrhea and associated fluid loss and dehydration, which may result in body weight loss. The present model, as expected and observed in previous studies [20,21,23,26], induces moderate diarrhea without weight loss in neonatal rats. In an attempt to ascertain whether this effect was not observed due to the male and female pooling of results, animals were properly sex-stratified, but again no differences were found. However, we cannot discard the possibility that this effect could also be differentially presented in males and females and this should be further studied in some RV diarrhea animal models with more virulent strains that induce serious fluid loss and affect body weight [29]. In this sense, there is non-available data in humans.

With regard to incidence, we observed that the maximum percentage of animals displaying diarrhea tended to be lower in females (*p* < 0.06), but overall, no statistical differences were found due to sex after the experimental inoculation. In spite of this tendency, the lack of evidence is in line with other works showing similar incidences of RV infection in male and female human individuals [30,31,32]. However, there are some studies indicating higher prevalence of RV infection in boys than in girls [33,34] and in some cases with a very clear boys-to-girls infection ratio of 1.8:1 [35]. In contrast, a higher susceptibility to RV infection has also been reported in female buffalo calves compared to males [36]. In addition, in our study we observed a similar duration of diarrhea between sexes. To our knowledge, there are no studies about the differential duration of diarrhea between males and females during RV infection either in humans or in animal models. Overall, although without statistical significance, our results reinforce the idea of higher susceptibility to infection in males which can be related to the lower humoral and cellular immune responses [1,2,3,4].

In children with RV infection, the most common clinical features are diarrhea, vomiting, fever and dehydration. There is a variety of severity classification systems, such as hydration status, the Vesikari Clinical Severity scoring system or the need for hospitalization. Therefore, it is difficult to compare the severity of RV infection between studies in children, making it more difficult to compare it with the rat model [37]. In the present work, males showed a 26 % increase in severity of diarrhea compared to females. Moreover, the severity of diarrhea was also observed by the fecal weight increase during the diarrhea period, which also displayed differences between sexes. Thus, both severity indicators, diarrhea index and fecal weight, demonstrated that males had more severe diarrhea than females. It is obvious that these differences are limited, but the agreement between a subjective measure—the score—and an objective one—the fecal weight—reinforces the result. In addition, our results are in agreement with other authors, which have suggested that male children suffer more from diarrhea than females, even if their results did not achieve statistical significance [38]. On the other hand, Muendo C. et al. did not observe differences in severity between boys and girls using the Vesikari Clinical Severity scoring system [37]. To our knowledge, this is the first experimental approach demonstrating differences in diarrhea severity between males and females in the context of RV infection. In the future, it would be important to properly record this type of information at a clinical level in order to be able to confirm these preclinical results.

It is widely accepted that females have a reduced susceptibility to viral infections because they immunoreact more strongly than males. This knowledge is in line with the lower severity found herein for female rats. However, it is also suggested that females, as a consequence of their higher active immune response are more predisposed to develop some immune-pathogenic effects (associated with higher inflammatory response involving chemokines and cytokines) of the viral infection and consequently to present more severe symptoms [39]. This rationale does not agree with the reduction in severity of the RV-diarrhea shown here. Thus, the mechanisms involved in controlling the disease such as the viral elimination and the antibody response merit further study.

In humans, as stated before, the etiological causative agent in gastroenteritis is not always identified and if it is, the viral elimination quantification is not the usual approach performed. In our controlled model of RV diarrhea, we have been able to quantify the RV particles in feces as a reflection of the infection [20,21,23,26]. The sex-stratified analysis of SA-11 shedding showed that males had a higher viral load in feces compared to females the day after infection, a result that seems to be in line with other studies [35]. This value reflects how the organism is able to destroy and eliminate the virus in the early phase, when the peak is maximum. Thus, this result indicates that females were able to more efficiently control the virus, maybe by triggering mechanisms that reduce the adhesion of the virus to the host. Viruses blocked by maternal IgA are not detected by this technique, however the variability in this load provided by the lactating dam cannot be a confounding factor, because each litter was composed of a similar number of females and males. In addition, the level of plasma anti-RV Ig in gestating dams was also checked showing similar results between siblings, independently of their sex.

In women, higher levels of Igs and specific Igs against viruses and vaccines have been described [6]. Thus, we wanted to study whether the clinical variation observed was due to a different immune response (i.e. Ig and anti-RV titers). No differences were appreciated in the levels of overall Ig in basal conditions on 11 DPI. Conversely, the pattern of Ig changed in RV-infected animals displaying a higher Th1/Th2 ratio associated with Ig. This change was mainly due to a reduction in IgG1, a Th2 associated Ig, for both males and females and also to a reduction in IgG2a, which was only observed in females. This differential Ig pattern between reference and infected animals has been previously observed when considering both males and females together [40]. It has to be taken into account that the Ig-enriched maternal milk strongly influences the pups’ plasma Ig levels, as observed in previous studies [41]. Therefore, the developing immune sexual dimorphism in early life [42], such as this punctual dimorphism in IgG2a levels in the infected groups can be masked by the Ig absorbed from breast milk in this period. Regarding the specific response, the levels of anti-RV antibodies on 11 DPI indicated that the Ig profile after the resolution of the infection was similar between sexes. Indeed, the anti-RV adaptive response at that stage is probably low or absent, because of a still developing and immature immune system [43]. In line with this, the fact that siblings displayed similar levels of Ig subtypes and subclasses and anti-RV Ab levels, and clustered together in the PCA, reinforces the idea that passive breast milk transfer is critical in the immune response against the virus. This is further reinforced by the correlation of anti-RV Ab levels in breast milk and those in pups. However, the transplacental route should also be taken into account. To our knowledge, this is the first study evaluating the sex-associated immune response against RV, both in animal models or in humans and its influence on passive immunity. However, it would have been interesting to asses, rather than just Ab levels, their neutralization capacity against the virus, in order to better explore the role of sex in the anti-viral response.

In our results, the humoral immune response was not different between sexes, thus some other mechanisms should explain the lower incidence and severity of diarrhea found in females. In fact, besides adaptive immunity, innate immunity has also been observed to be sex-associated [44,45]. This led us to explore whether the local innate defense strategies such as intestinal barrier function (mucin production or tight junction protein levels) and TLR were involved. In this regard, previous studies with this model have shown that RV-infected animals increased mucin production [21] and TLR activation [46] as a response to injury. In our case, the gene expression of those molecules at 3 DPI (at very early age and during the peak of diarrhea) was not sex-associated, but on 11 DPI (in a later stage of development and after infection resolution), besides many tendencies that should be confirmed, higher mucin and TLR9 gene expression was found. It still remains to be confirmed whether these innate mechanisms are subjected to sexual dimorphism and, if so, whether they are, at least in part, responsible for the differential clinical outcomes.

Overall, the mechanisms involved in the sexual dimorphism against viral infections are poorly understood. As we mentioned before, the X chromosome plays an important role in regulating the immune function and the Y chromosome also contains regulatory response genes [4]. For this reason, the dimorphism observed in the immune response could result from the direct effects of the sex chromosomes or from their indirect effects, such as from sex hormones [47]. For this reason, a careful examination of X-linked genes would have also been helpful in terms of explaining the mechanisms involved in the differences in the RV-symptoms found here.

In addition, it has been demonstrated that gut microbiota also differ between sexes [10,12,47,48,49,50,51,52], although the timing in which this dimorphism appeared remains unclear [53]. The interaction between the intestinal microbiota and the intestinal immune system can be reciprocal and each can influence the other. Previous studies in humans and in our rat model have also shown a disbiosis associated with the infection and have shown how the modulation of the microbiota composition is associated with clinical amelioration [46]. Specific bacterial strains and particular prebiotics have been shown to improve RV gastroenteritis by both direct and indirect mechanisms, such as pathogen aggregation or immunomodulation, respectively [25,28]. Therefore, gut microbiota sexual dimorphism should also be addressed in future studies. 

In addition, the two live attenuated oral vaccines, RotaTeq (Merck and Co, West Point, PA, USA) and Rotarix (GSK Biologicals, Rixensart, Belgium), have shown safety and efficacy in children but, the existing studies have not addressed properly the different responses in boys and girls [54,55,56]. Thus, these data lead us to suggest an interest in further studying this response in the context of vaccination and prevention of future reinfections.

## 5. Conclusions

Our animal study demonstrates that the susceptibility and the host response to viral infections are slightly different between sexes. Many studies of human and rodent viral infections use only one sex or do not report the sex of their subjects. The results obtained without checking the possible influence of the sex could lead to wrong conclusions. For this reason, the inclusion of sex as a criterion will improve the understanding of the sex-specific differences in the response to infections and the development of treatments against viral infections. 

## Figures and Tables

**Table 1:** Clinical variables determining the diarrhea process in the RV group.

Clinical Outcome	Variable	Males (n = 39)	Females (n = 57)
Incidence	MDA	53.85%	56.14%
	MDAd	3 DPI	3 DPI
	daAUC	174.36	184.21
	MDF	86.67%	72.73%
	MDFd	1 DPI	3 DPI *
	dfAUC	285.93	252.57
Duration	DDB	1.78 ± 0.16 DPI	2.05 ± 0.17 DPI
	DDE	3.37 ± 0.21 DPI	3.45 ± 0.16 DPI
	DP	1.48 ± 0.24 DPI	1.28 ± 0.19 DPI
	DwD	2.03 ± 0.21 DPI	2.00 ± 0.17 DPI
Severity	MDI	2.49 ± 0.09 *	2.28 ± 0.06
	MDId	2.26 ± 0.20 * DPI	2.76 ± 0.14 DPI
	sAUC	4.53 ± 0.35 *	3.76 ± 0.21

Results are expressed as mean ± SEM (n = 39–57 animals/group). MDA, maximum percentage of diarrheic animals; MDAd, day with maximum percentage of diarrheic animals; daAUC, area under the curve of diarrheic animals; MDF, maximum percentage of diarrheic feces; MDFd, day with maximum percentage of diarrheic feces; dfAUC, area under the curve of diarrheic feces. DDB, day of diarrhea beginning (DPI); DDE, day of diarrhea ending (DPI); DP, diarrhea period; DwD, days with diarrhea. MDI, maximum diarrhea index; MDId, day of maximum diarrhea index (DPI); sAUC, area under the curve of severity. Statistical differences: *: *p* < 0.05 males vs. females.

**Table 2:** Concentration of immunoglobulins (mg/mL) in plasma at the end of the study (11 DPI).

Immunoglobulins	REF Males	REF Females	RV Males	RV Females
IgA	18.5 ± 1.82	38.0 ± 9.5	47.7 ± 1.74	46.0 ± 11.3
IgM	23.6 ± 2.73	22.3 ± 1.43	21.9 ± 1.72	20.4 ± 0.75
IgG	3070 ± 520	3400 ± 191	3820 ± 895	3610 ± 498
IgG1	279 ± 30.1	239 ± 28.4	159 ± 21.0 *	150 ± 11.4 *
IgG2a	549 ± 67.6	648 ± 60.6	512 ± 65.9	492 ± 45.4 *
IgG2b	1180 ± 207	1280 ± 99.2	2090 ± 813	1930 ± 45.2
IgG2c	1070 ± 252	1230 ± 1114	1060 ± 126	1040 ± 67.7
Th1/Th2 ^#^	2.61 ± 0.29	2.88 ± 0.20	4.55 ± 0.93 *	4.56 ± 0.52 *

Results are expressed as mean ± S.E.M (n = 6–14 animals/group). Statistical differences: * *p* < 0.05 RV males vs. REF males or RV females vs. REF females. ^#^ Th1/Th2 ratio refers to the relationship between Th1 (IgG2b + IgG2c) and Th2 (IgG1 + IgG2a) immunoglobulins.
